# Determination of Heterogeneous Proteomic and Metabolomic Response in anti-TNF and anti-IL-6 Treatment of Patients with Rheumatoid Arthritis

**DOI:** 10.3390/life13020596

**Published:** 2023-02-20

**Authors:** Alexander A. Stepanov, Kristina A. Malsagova, Arthur T. Kopylov, Vladimir R. Rudnev, Dmitry E. Karateev, Evgenia I. Markelova, Elena L. Luchikhina, Elena E. Borisova, Anna L. Kaysheva

**Affiliations:** 1 Biobanking Group, Branch of IBMC “Scientific and Education Center”, 109028 Moscow, Russia; 2 Moscow Regional Research and Clinical Institute (“MONIKI”), 129110 Moscow, Russia

**Keywords:** metabolome, proteome, rheumatoid arthritis, HPLC-MS/MS, inhibitors of TNF, inhibitors of IL6, ELISA

## Abstract

Reduction in tumor necrosis factor (αTNF) and interleukin-6 (IL-6) activities is a widely utilized strategy for the treatment of rheumatoid arthritis (RA) with a high success rate. Despite both schemes targeting the deprivation of inflammatory reactions caused by the excessive activity of cytokines, their mechanisms of action and the final output are still unequal. This was a comparative longitudinal study that lasted for 24 weeks and aimed to find the answer to why the two schemes of therapy can pass out of proportion in attitude of their efficiency. What are the differences in metabolic and proteomic responses among patients who were being treated by either the anti-TNF or anti-IL-6 strategy? We found increased levels of immunoglobulins A and G (more than 2-fold in anti-IL-6 and more than 4-5-fold in anti-TNF groups) at the final stage (24 weeks) of monitoring but the most profound increase was determined for µ-chains of immunoglobulins in both groups of study. Metabolomic changes displayed main alterations with regard to arginine metabolism and collagen maintenance, where arginine increased 8.86-fold (*p* < 0.001) in anti-TNF and 5.71-fold (*p* < 0.05) in anti-IL-6 groups but patients treated by the anti-TNF scheme suffered a higher depletion of arginine before the start of therapy. Some indicators of matrix and bone tissue degradation also increased 4-hydroxyproline (4-HP) more than 6-fold (*p* < 0.001) in anti-TNF and more than 2-fold (*p* < 0.05) in the anti-IL-6 group, but the growth dynamics in the anti-IL6 group was delayed (gradually raised at week 24) compared to the anti-TNF group (raised at week 12) following a smooth reduction. The ELISA analysis of IL-6 and TNFα concentration in the study population supported proteomic and metabolomic data. A positive correlation between ΔCDAI and ΔDAS28 indicators and ESR and CRP was established for the majority of patients after 24 weeks of treatment where ESR and CRP reduced by 20% and 40% finally, respectively. A regression model using the Forest Plot was estimated to elucidate the impact of the most significant clinical, biochemical, and anthropometric indicators for the evaluation of differences between considered anti-TNF and anti-IL-6 schemes of therapy.

## 1. Introduction

The molecular profile of patients with rheumatoid arthritis (RA) reflects the change in the level of endogenous molecules during therapeutic interventions. In this regard, understanding such changes allows for clarifying the pathogenesis of the disease. Some therapy agents are targeting tumor necrosis factor-α (TNF-α), which is a pro-inflammatory cytokine and plays a key role in cell metabolism of glucose and lipids [1]. It is likely that the intervention of TNF-α inhibitors (TNFis) into therapeutic practice causes a change in the metabolic profile [2].

The anti-TNF-α therapy affects key cellular metabolic processes, including the tricarboxylic acid cycle, and amino acid and fatty acid metabolism. The analysis of urine samples of those patients with RA receiving etanercept and infliximab for six months displayed an increased level of histamine, glutamine, phenylacetic acid, xanthine, xanthurenic acid, and creatinine, and a decrease in ethanolamine, hydroxyphenyl pyruvic acid, and phosphocreatine compared to baseline [3]. The TNF antagonizing therapy also revealed an association between urinary metabolites and the disease activity amongst responding patients unlike non-responding subjects [3].

Reversed-phase liquid chromatography (RP-UHPLC) and quadrupole-time-of-flight mass spectrometry (ESI-QTOF-MS) of plasma samples of RA patients who were treated by infliximab, abatacept, and etanercept indicate the prospect of detecting carbohydrate derivatives (D-glucose, D-fructose, sucrose, and maltose) as candidate metabolites to classify responding and non-responding subjects [4]. Plenty of studies are devoted to the identification of featured metabolites using nuclear magnetic resonance (1H-NMR), which show changes in the level of certain circulating amino acids (isoleucine, leucine, valine, alanine, glutamine, tyrosine), sugars, and fatty and carboxylic acids in response to TNFi therapy [2,5,6,7,8].

Monoclonal antibody tocilizumab, specific to interleukin-6 (IL-6), interferes with IL-6 signaling and shows potential therapeutic value in the treatment of RA. IL-6 is believed to influence lipid metabolism by promoting lipid uptake through induction of very low-density lipoprotein (VLDL) and the increase in lipolysis in liver and adipose tissue, thus reducing the lipid production in liver [5]. Serum triglycerides (TG), total cholesterol (TC), and HDL-C levels are reported to be elevated, when exposed to anti-IL-6 agents [6,7]. It should be noted that the effect on the atherogenic index is indirect, as various studies show an increase in LDL-C by 15–20% [7].

Metabolites of patients with the early-diagnosed RA have been associated with sustained drug-free remission (DAS28 < 2.6) after tocilizumab (TCZ) or methotrexate (MTX) therapy [8]. A study using TCZ showed that increasing levels of 3-hydroxybutyrate and phenylalanine may improve the ability to predict patients responding to TCZ therapy [9]. In addition, TCZ treatment may modulate arachidonic acid metabolism by affecting IL-6 signaling [10]. Tryptophan, which is a substrate for the enzyme indolamine 2,3-dioxygenase (IDO2), has been shown to be required for CD4+ T-cells activation and autoantibody production and may play a role in the development of arthritis in a mouse model [11].

In addition to monitoring metabolite changes in response to treatment, the severity of symptoms has also been associated with pain. Pain, the dominant component of patient-reported outcomes, is present in nearly one-third of patients after 21 months of the combinatory therapy [12].

Given the increasing recognition of the role of the JAK/STAT pathway and its blockers (tofacitinib/baricitinib) in modulating pain and the nociceptive response, this pathway and nociceptive cytokine signaling have been observed in rheumatoid arthritis and psoriatic arthritis [12]. Tofacitinib and baricitinib (JAK inhibitors) have been shown to increase levels of omega-3 fatty acids and docosahexaenoic acid (DHA) in treated patients, which has been associated with a significant reduction in pain [13]. Thus, several studies using TNF inhibitors have observed modulations of tryptophan, valine, leucine, lysine, creatinine, and alanine in biological samples, whereas JAK/STAT inhibitors can only modulate fatty acids.

The aim of this study was to identify molecular patterns (proteins and endogenous metabolites) specified for three types of RA therapy: inhibitors of IL6, TNF, and Janus kinases.

## 2. Materials and Methods

### 2.1. Reagents

Urea (99%) and formic acid (98%+, pure) were obtained from Acros Organics (Geel, Belgium). Trifluoroacetic acid (99%, Reagent Plus^®^), triethylammonium bicarbonate (1 M solution), 4-vinylpyridine (95%), and sodium deoxycholic acid (>97% titration) were from Sigma (St. Louis, MO, USA). Acetonitrile (HPLC grade, filtered for 0.2 µm) was purchased from Fisher Chemical (Loughborough, UK). Acetic acid (EMSURE^®^, glacial, anhydrous for analysis) was from Merck (Darmstadt, Germany). TCEP (Tris(2-carboxyethyl) phosphine hydrochloride) was purchased from Pierce™ (Thermo Fisher, Rockford, IL, USA). Trypsin (sequencing-grade-modified), 5 vials × 20 µg, lyophilized powder, were from Promega (Madison, WI, USA). β-glucuronidase from the Escherichia coli K12 strain, 5 mL falcon vial, specific activity ~140 U/mg at 37 °C, was from Roche Diagnostics GmbH (Mannheim, Germany). Water (TOC < 3 ppb) was obtained from the Milli-Q Integral 3 purification system, Millipore S.A.S (France). Oasis^®^, 3 cc (cubic centimeter) nominal volume, 60 mg resin, type of resin: WAX-weak anion exchange, was from Waters Corporation (Milford, MA, USA).

### 2.2. Ethical Consideration

The study design was approved by the local Ethical Committee of the Moscow Regional Clinical Research Institute named after M.F. Vladimirsky (MONIKI) Protocol no.18 (24 December 2020). All handlings and uses of material were provided according to the WMA Declaration of Helsinki on Ethical Principles for Medical Research Involving Human Subjects (revision Fortaleza, Brazil, 2013).

### 2.3. Subjects

The study involved 40 patients diagnosed with rheumatoid arthritis. The annotation of biological samples contained the following information (Table 1):

(1) Sample information: unique identifier of the study participant and type of drug therapy;
(2) Information about the anthropometric characteristics of the participant: gender, height, weight, body mass index, age at the time of the examination;
(3) Information about the clinical and medical characteristics of the participant: diagnosis or code ICD-10, Steinbroker stage, concomitant diseases, history of therapy of the underlying disease, drug prescribed in the study, duration of the disease (months), assessment of disease activity by DAS28-ESR indices, assessment of disease activity by CDAI indices, assessment of functional status by HAQ index;
(4) Information on clinical biochemical parameters of blood: the level of C-reactive protein (CRP), erythrocyte sedimentation rate (ESR), CCP (cyclic citrullinated peptide) antibodies.

### 2.4. Preanalytical Stage for Proteomic Analysis

The analyzed blood plasma sample with a volume of 100 µL was transferred into clean labeled tubes; a solution of 85% orthophosphoric acid with a volume of 6 µL was added (up to 5% in the final concentration; Sigma, Germany) and mixed; methanol was added with a volume of 800 µL (J.T. Baker, Landsmeer, The Netherlands) and mixed again. The resulting suspension was centrifuged at 10,000 rpm at 15 °C for 10 min (Centrifuge 5424R, Eppendorf, Germany). The resulting precipitate was reconstituted with a denaturing solution in a volume of 50 µL (solution composition: 5 M urea (Sigma, Germany), 1% deoxycholic acid sodium salt (Sigma, Italy), 300 mM sodium chloride (Fluka-Honeywell, Germany), 10% acetonitrile (Carlo Erbo, Val-de-Reuil, France), 100 mM triethylammonium bicarbonate, pH 8.2–8.5 (Sigma, Buchs, Switzerland), to which 10 μL of a 50 mM solution of neutralized TCEP (tris-(2-carboxyethyl)phosphine; Sigma, St. Louis, MO, USA) was added up to 10 mM at final concentration. The reconstituted denatured protein solution was incubated at 450 °C for 30 min with constant vigorous stirring at 1200 rpm (Thermo Mixer, Eppendorf, Hamburg, Germany). Then, 6 μL of a 2% solution of a stabilized solution of 4-vinylpyridine (Aldrich, Gillingham, UK) in a solution of 30% isopropanol (Fisher Chemical, Loughborough, UK) to 0.2% at final concentration was added. The alkylation reaction was incubated out of light at room temperature for 20 min. At the end of the reaction, the sample volume was adjusted to 500 µL by adding 384 µL of a 75 mM triethylammonium bicarbonate solution (pH 8.2) and thoroughly mixed. An amount of 400 ng of trypsin at a concentration of 100 ng/µL (Promega, Madison, WI, USA) in a solution of 30 mM acetic acid (Carlo Erba, Val-de-Reuil, France) was added to each sample and incubated for 3 h at 40 °C with intermittent stirring (every 10 min at 1700 rpm for 90 s). An additional aliquot of 400 ng of trypsin (100 ng/µL) in 30 mM acetic acid was then added and reacted for a further 2 h at 42 °C, with interval stirring (every 10 min at 1700 rpm for 90 s). At the end of the incubation time, 10 μL of absolute formic acid was added and the precipitation of the reduced deoxycholic acid was observed. The resulting suspension was centrifuged at 12,000 rpm at 10 °C for 10 min. The supernatant (500 µL) was taken and an equal volume (500 µL) of ethyl acetate (Carlo Erba, France) was added to it to remove the residual amount of deoxycholic acid by vigorous stirring for 3 min at room temperature. Then, the sample was centrifuged at 8000 rpm at 20 °C for 5 min and the sample was frozen at −20 °C for 10–15 min. Samples were taken from the freeze mode, the surface organic layer was drained, and 150 µL of acetonitrile (Carlo Erba, France) was added to the aqueous solution of peptides. It was centrifuged at 13,000 rpm at 20 °C for 10 min, and the supernatant was collected and dried under vacuum at 30 °C for 60–70 min with occasional ventilation of the chamber every 15 min (Concentrator Plus, Eppendorf, Germany). The resulting dry residue was reconstituted in 20 μL of 0.5% formic acid (Sigma Germany).

### 2.5. Proteomic Analysis

Analysis was performed on a Xevo™ G2-XS Q-tof quadrupole time-of-flight mass spectrometer (Waters, Wilmslow, UK) coupled to an Acquity™ UPLC H Class Plus chromatography system (Waters, UK). The analysis was carried out in the mode of positive electrostatic ionization in the increased sensitivity mode of the mass analyzer with the normal dynamic range of mass registration. The emitter voltage was 3 kV, the drying gas velocity was 680 L/min, the focusing gas velocity was 50 L/min, the ionization source temperature was 150 °C, and the desolvation temperature was 350 °C. The focusing cone voltage was 67 V with a bias of up to 130 V. The ions were recorded in the hybrid information-independent (DIA) MS^E^-SONAR mode. The primary information-independent (DIA) MS scan was performed in the range of 100–1500 m/z, followed by scanning in SONAR mode with quadrupole mass isolation in the range from 400 m/z to 1100 m/z with an isolation width of 22 Daltons. The total time for one complete scan cycle was set at 0.418 s. Fragmentation was performed in a two-phase mode: phase 1—low-energy CID fragmentation with argon at 6 eV; phase 2—high-energy graded CID fragmentation with argon in the range from 15 to 37 eV. During the assay, active mass correction m/z = 556.27 with low activation energy (9 eV) according to the Leu-enkephalin standard (50 pg/mL in 50% acetonitrile with 0.1% formic acid) was used with injection at a speed of 5 µL/min for 30 ms into the ionization source at intervals of every 45 s and isolation within 200 mDa.

Chromatographic separation was performed on an Acquity™ UPLC BEHC18 column (1.7 µm particle size, geometry 2.1 × 50 mm; Waters, UK) at a flow rate of 0.2–0.3 mL/min and constant incubation at 40 °C of phase A (aqueous solution of 0.1% formic acid and 0.03% acetic acid) and phase B (solution of 0.1% formic acid and 0.03% acetic acid in acetonitrile) with the following elution scheme: 0–1.5 min—3% B, up to 26.5 min—19% B, up to 42 min—32% B, and up to 43.5 min—97% B, which was held in isocratic mode up to 47.5 min, with a decrease up to 3% B until the 49th minute and then held in isocratic mode up to 53 min. The flow rate in the interval 43.5–47.5 min was 0.3 mL/min, and that in all other cases was 0.2 mL/min.

The analysis of the obtained data was carried out in the PLGS program (Protein Lynx Global Server, version 3.0.3, Waters, UK) using the UniProt KB database (version dated March 2021) with preset parameters for the SONAR/MS^E^ scanning mode and preset correction for the calibration mass.

### 2.6. Preanalytical Stage for Metabolomic Analysis

Total metabolic fractions were recovered using liquid- and solid-phase extraction. To this end, 100 µL of plasma was enriched with 10 µL of deuterated d5-betamethasone in methanol (catalog number B327002, Toronto Research Chemicals; Toronto, ON, Canada) as an internal standard to 10 ng/mL at the final concentration. Plasma was diluted with ice-cold methanol to 400 μL for deproteinization. Samples were stirred vigorously for 10 min at 15 °C and then centrifuged at 10 °C for 15 min (10,000 rpm) to precipitate proteins. The supernatant was collected in a volume of 300 µL and transferred to a new clean glass labeled tube. The deproteinized plasma solution was diluted to 3 mL with 50 mM sodium phosphate buffer, pH 6.3. Then, 50 µL of β-glucuronidase from the Escherichia coli K12 strain (Roche Diagnostics GmbH; Germany) was added to the resulting solution and incubated for 1 h at 55 °C. Hydrolysis of the glucuronic conjugates was stopped by adding 15 mM potassium carbonate, pH 9.0. An amount of 1.5 mL of ethyl acetate with n-pentane (9:1, *v*/*v*) was added to the samples and shaken vigorously for 5 min at 20 °C, then left at −20 °C in the refrigerator for 15 min. The organic layer was transferred to clean labeled tubes and the extraction procedure was repeated. The collected fractions were combined and dried under nitrogen flow. The aqueous phase was transferred to a WAX cartridge (weak anion exchange carrier; 60 mg, Oasis™ series, Waters, UK) for solid-phase extraction, pre-equilibrated with 3 mL of 2% ammonium hydroxide solution and twice with 3 mL of water. The metabolites were washed successively with 3 mL of water and eluted twice in 3 mL of 2% formic acid in methanol. The eluted fractions were combined and dried in vacuum at 45 °C.

### 2.7. Metabolic Analysis

The metabolome extracted from plasma (preanalytical stage) was analyzed using a Xevo™ G2-XS Q-tof high-resolution quadrupole time-of-flight mass spectrometer (Waters, UK) coupled to an Acquity™ HPLC H Class Plus chromatography system (Waters, UK) in positive mode with ionization with a static mass registration range within 50–800 m/z. The emitter voltage was 2.4 kV, the drying gas velocity was 700 L/min, the focusing gas velocity was 50 L/min, the temperature in the ionization source was 150 °C, and the temperature in the desolvation was 440 °C. The voltage at the focusing cone was 15 V with a bias of up to 26 V. Ions were registered in the information-dependent (DDA) mode. Initial information-dependent scanning was carried out in the range of 50–800 m/z, then scanning was performed in tandem mode with mass isolation by a quadrupole in the range from 50 m/z to the mass of the precursor ion with an increment of 20 atomic mass units. The total time for one complete scan cycle was set at 0.192 s. Fragmentation was carried out in a two-phase mode: phase 1—low-energy CID fragmentation with argon at 12 eV, phase 2—high-energy graded CID fragmentation with argon in the range of 20–45 eV. Active weight adjustment m/z = 556.27 with low-activation-energy (9 eV) Leu-enkephalin standard (50 pg/mL in 50% acetonitrile with 0.1% formic acid) was used during the assay with an injection at a rate of 5 µL/min for 30 ms into the ionization source at intervals of every 45 s and isolation within 200 mDa.

Chromatographic separation was performed on a Zorbax™ RRHD C18 column (2.8 µm particle size, geometry 2.1 × 50 mm; Agilent, Santa Clara, CA, USA) at a flow rate of 0.4 mL/min and constant temperature control at 50 °C in a gradient of mobile phase A (aqueous solution of 0.1% formic acid and 0.005% heptafluorobutyric acid) and phase B (solution of 0.1% formic acid and 0.005% heptafluorobutyric acid in a mixture of acetonitrile and methanol in a 7:3 volume ratio) with the following elution scheme: 0-0, 5 min—2% B, up to 2.5 min—10% B, up to 5 min—18% B, up to 10.5 min—39% B, up to 13 min—42% B, and up to 14 min—98% B, which was held in isocratic mode for up to 16 min and decreased to 2% B by the 17.5th minute, followed by retention in isocratic mode for up to 20 min.

### 2.8. Data Analysis

For proteomic and metabolomic datasets, the Wilcoxon test with Bonferroni adjustment were applied. Beta coefficients for the forest plot were obtained in the multiple regression model that relates a DAS28 at week 24 corrected to baseline (response variable) to two predictor variables—baseline DAS28 and a protein or a metabolite. Statistical analysis was performed using R (v4.1.2; R Core Team 2021).

## 3. Results

### 3.1. Proteome Analysis

We examined 231 files obtained as a result of HPLC-MS/MS analysis of plasma samples of three groups of patients with RA receiving two therapy options: anti-TNF or anti-IL. The summed size of the proteome before the appointment of a certain therapy strategy was 884 proteins. In the anti-αTNF group, the proteome size was 554 proteins, and in the anti-IL6 group, it was 763 proteins. Proteins specific to the certain group of study made up a large proportion of the total proteome. Thus, 470 protein identifications were specific before the treatment, 384 proteins for the group of patients with anti-IL6 therapy, and 260 proteins for patients with anti-αTNF therapy. In anti-TNF therapy, the largest number of proteins were identified as specific before the start of treatment (n = 677), while more modest results were obtained after two weeks (n = 224), four weeks (n = 94), and 12 weeks (n = 10) of treatment. For anti-IL6 therapy, a specific group of n = 594 protein identifications were identified before the start of therapy; two weeks later, n = 207 proteins; after four weeks, n = 53; after 12 weeks, n = 122 proteins; after 24 weeks of treatment, a group of n = 64 specific proteins (Table 2). In the vast majority of cases (up to 90%), a linear range was detected. Among the identified proteins shared between all groups of study, 23 proteins were eventually selected (Table 2).

**Figure 1:**
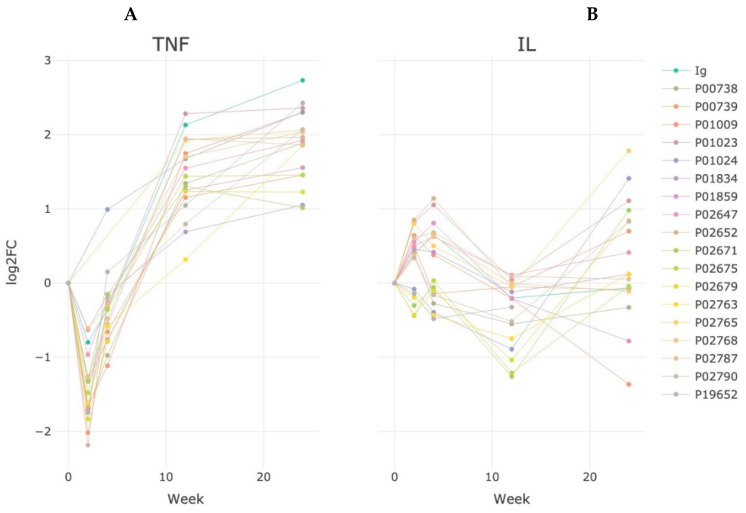
Dynamics of semiquantitative changes in the proteome of patients in the anti-αTNF (**A**) group and in the group with anti-IL6 (**B**) therapy during 24 weeks of treatment to baseline values before the start of therapy. The log2FC values for Ig reflect the fraction of proteins of the immunoglobulin class. Log_2_FC is calculated as a ratio of the median of Ig-proteins quantity toward its quantity at the initial point (before the starting treatment).

Immunoglobulins A and G (various isoforms of immunoglobulin light and heavy chains) dominated among the differentially expressed proteins both in the anti-αTNF and anti-IL6 groups of patients. The most significant differences can also be noted for µ-chains, which are specific for M-immunoglobulins (Figure 1).

In the case of anti-TNF therapy, an increase in immunoglobulins was achieved already at the 12th week of observation and then an exit to the steady state was observed, while in patients with anti-IL6 therapy, there was a delay in the development of the response, as after 12 weeks of treatment, most of the antibodies were close to the baseline (before treatment) and only by the 24th week did they start gradually dominating among other proteins (Figure 1 and Table 2). Among the patients of the anti-IL6 therapy group, we noted an almost twofold increase (*p* < 0.05) and, among the patients of the anti-TNF therapy group, a fourfold increase of hemopexin after 24 weeks of treatment (*p* < 0.05). 

**Figure 2:**
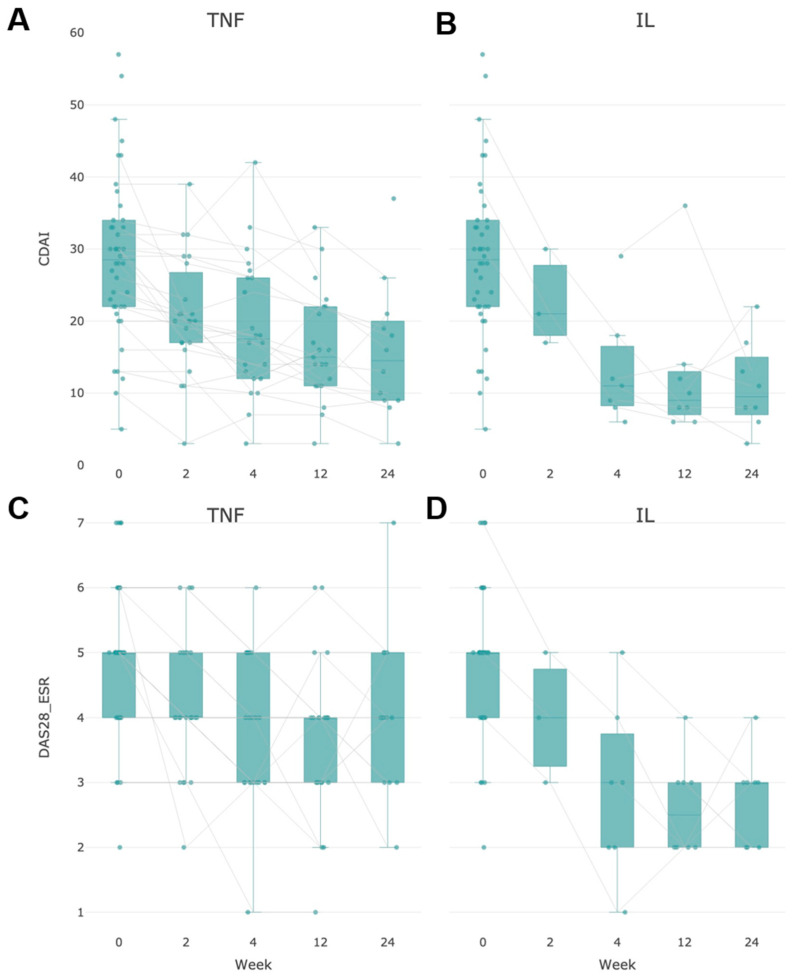
Change in clinical ΔCDAI in the group of patients with anti-αTNF (**A**) and anti-IL6 therapy (**B**) and ΔDAS28-ESR in the group of patients with anti-αTNF (**C**) and anti-IL6 (**D**) therapy for 24-x weeks of treatment. DAS28-ESR is used to determine eligibility for biologic therapies. DAS28-ESR ≥ 3.2 can be used as a threshold for classifying active RA.

A centralization of changes in the proteome around the same class of proteins was observed in both groups of study. In general, one can note the positive correlation of DAS28 and CDAI indicators with a lowering of the disease activity or remission during the observation in both the anti-TNF and anti-IL6 group, respectively (Figure 2). According to the observational data, the remission of low activity (DAS28 < 3.2) was already reached at week 12 and maintained at week 24, but the response effect and the proportion of patients with amelioration were higher in the group of anti-IL-6 therapy, which is illustrated by both CDAI and DAS28 scales (Figure 2).

**Figure 3:**
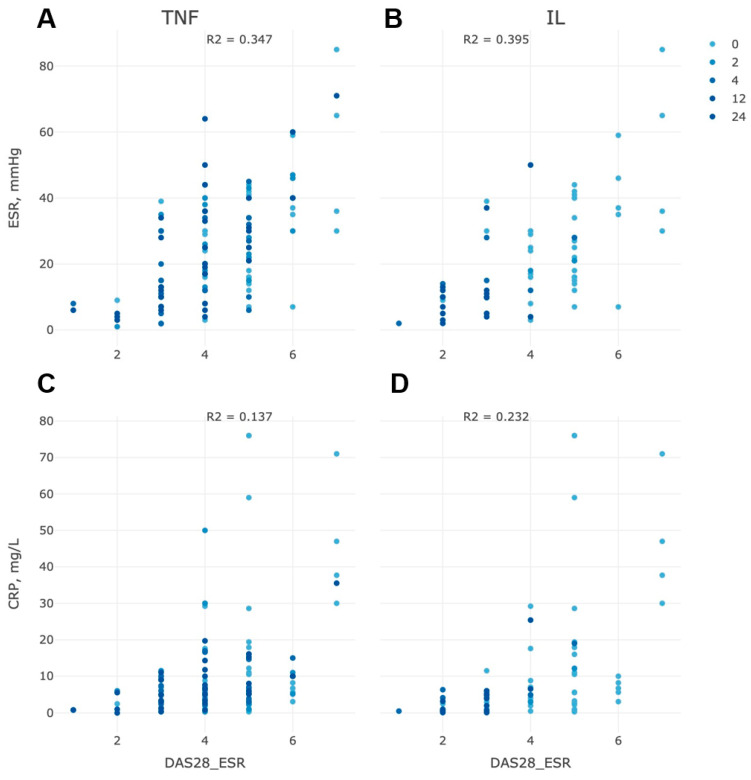
Correlation between ΔDAS28 and ESR in patients with anti-αTNF (**A**) and anti-IL6 (**B**) therapy and between ΔDAS28 and CRP in the group of patients with anti-αTNF (**C**) and anti-IL6 (**D**) therapy during 24 weeks of treatment.

We also noticed a pronounced positive correlation between ΔDAS28, CRP, and ESR indicators (Figure 3). The positive effect of the prescribed therapy scheme was signified by a simultaneous decrease in CRP and ESR by 40% and 20%, respectively, during the entire treatment history, which was reflected in the gradient decrease of DAS28 (Figure 3).

According to DAS28-ESR, the proportion of patients in remission or low activity of the disease (less than 3.2) was higher compared to the DAS28-CRP indicator at the end of the study. Thus, the agreement between DAS28-CRP and DAS28-ESR in categorizing patients with remission or low activity of the disease regardless of the type of therapy was nearly 27%–39%. Nevertheless, both indicators (DAS28-ESR and DAS28-CRP) showed no significant difference between types of therapy (anti-IL6 or anti-TNF).

**Figure 4:**
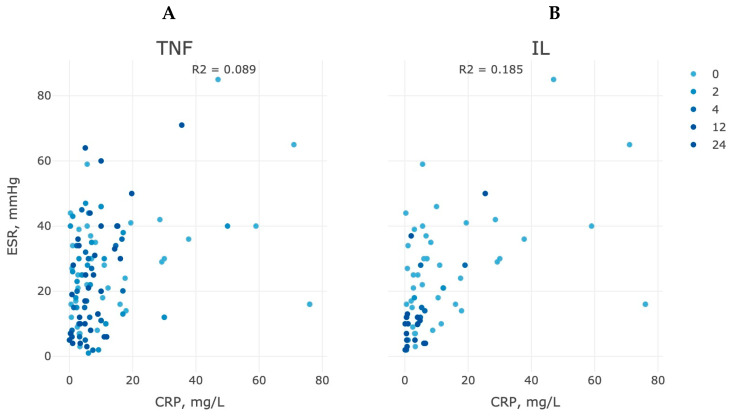
Inhibition of CRP production and the dynamics of the decrease in the erythrocyte sedimentation rate in patients of the anti-αTNF (**A**) and anti-IL6 (**B**) therapy groups during a 24-week course of treatment.

As DAS29 versions (ESR or CRP) were not interchangeable and often debatable in their discrepancy, we considered ESR vs. CRP separately for both types of therapy at the end of the study. The decrease in CRP levels with the ESR can, thus, strongly indicate the effectiveness of therapeutic treatment (Figure 4). Both groups of the study demonstrated a high proportion of patients with lowering CRP at week 24, which agreed with the DAS28-CRP version, but the group of anti-TNF therapy shared a denser proportion of patients (up to 70%) with a high ESR indicator (>10 mm/h) with a comparable value to anti-IL-6 CRP values.

### 3.2. Metabolome Analysis

**Figure 5:**
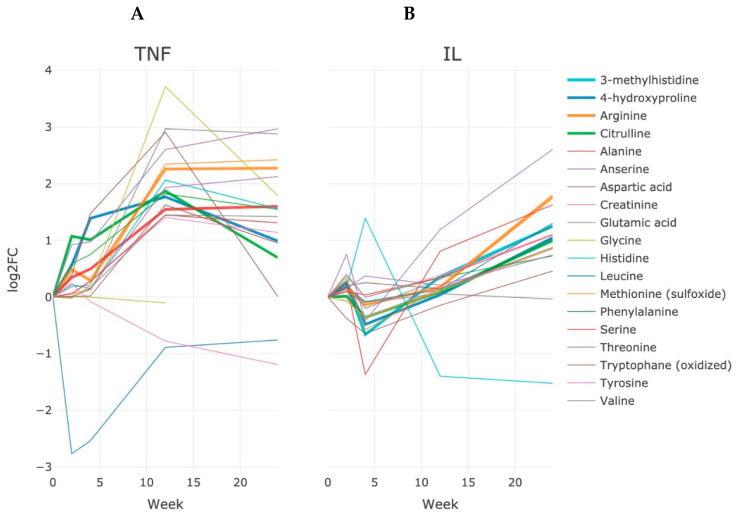
Dynamics of changes in endogenous metabolites during a 24-week course of treatment among patients in the anti-αTNF (**A**) and ani-IL6 (**B**) groups of therapy. The Log_2_FC value is calculated as a median quantity of a certain metabolite toward its quantity at the initial (before starting treatment) point of treatment.

The study of the metabolome was carried out with a focus on selected metabolites; however, the results of the study turned out to be less informative compared to the results of the proteomic study (Figure 5).

There was a more than sixfold increase (*p* < 0.001 after Bonferroni correction) of 4-hydroxyproline (4-HP) among patients with anti-TNF therapy relative to the baseline, which probably reflects the intensification of collagen resorption by action of osteoclasts. An increase of 4-HP was noted after 12 weeks of treatment, and thereafter, the level of 4-HP followed the unaltered state for the next 12 weeks (Figure 5). There was no more than a twofold (2.07, *p* < 0.05 with Bonferroni correction) increase in circulating 4-HP for 24 weeks of therapy in the group of patients with anti-IL6 therapy (Figure 5). A comparable situation persisted for 3-methylhistidine (3-MH), as an indicator of the destruction of muscle tissue and extracellular matrix. However, the dynamics was lower. Among patients with anti-TNF therapy, the concentration of 3-MH increased by more than three times (3.4, *p* < 0.05 with the Bonferroni correction by the 12th week of treatment); however, the level decreased twofold (1.98, *p* < 0.05 with Bonferroni correction) at week 24. Oddly, 3-MH levels did not rise more than twofold during the entire observation in patients with anti-IL6 therapy (Figure 5). In patients with anti-TNF therapy, the deficiency of arginine was observed before the start of therapy and it was completely replenished after 24 weeks of treatment (increase was more than 1.6-fold, *p* < 0.001), whereas in the anti-IL6 group, the increase in arginine was even more pronounced and reached 3.4 (*p* = 0.0059) relative to the baseline after 24 weeks. Meanwhile, in both groups of study along with arginine, the level of citrulline also increased (threefold, *p* < 0.001, in the anti-αTNF group and twofold in the anti-IL6 group, *p* > 0.05), indicating the normalization of arginine catabolism and a decrease in activity NOS2 synthase as the main mediator of inflammatory reactions.

### 3.3. Human IL-6 and TNF Immunoassay

**Figure 6:**
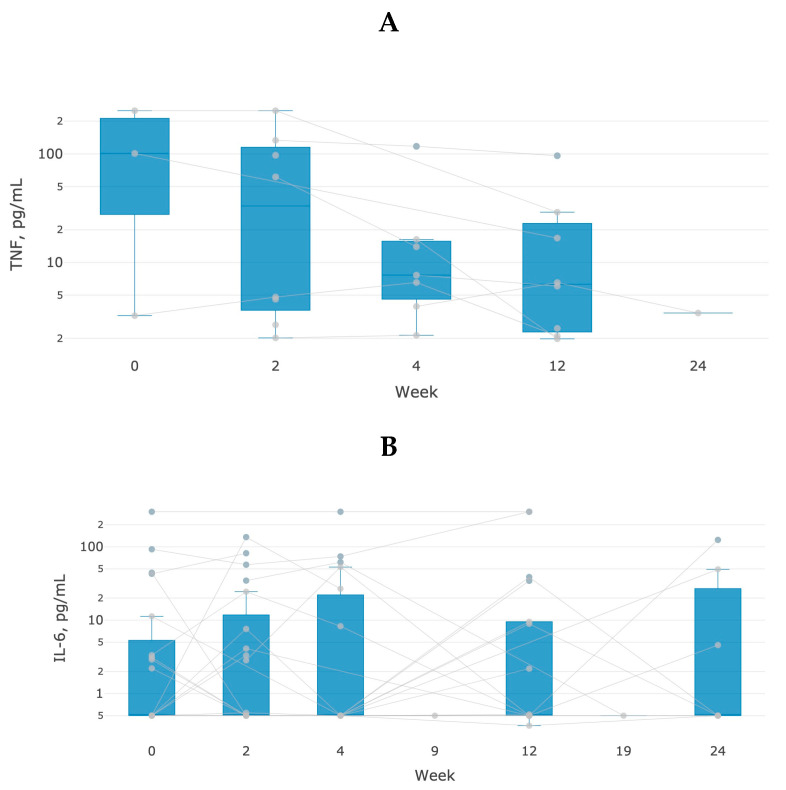
Dynamics of changes in the content of TNF (**A**) and IL6 (**B**) in blood samples of patients with anti-αTNF therapy. The measurements were performed by the ELISA method (Alfa-TNF-IFA-BEST and IL-6-IFA-BEST, Vector Best, Novosibirsk, Russia) in three technical repetitions.

The data of immunochemical analysis for IL6 and αTNF confirmed the results of proteome and metabolome assays (Figure 6). Among patients with anti-αTNF therapy, 11 patients had a twofold or more decrease in TNF (*p* < 0.05) at week 24, whereas among patients with anti-IL6 therapy, 40% of cases were characterized by a decreased level of interleukin-6 at week 24 of treatment (*p* < 0.05).

### 3.4. Association of Antibodies with anti-TNF and anti-IL6 Therapies

**Figure 7:**
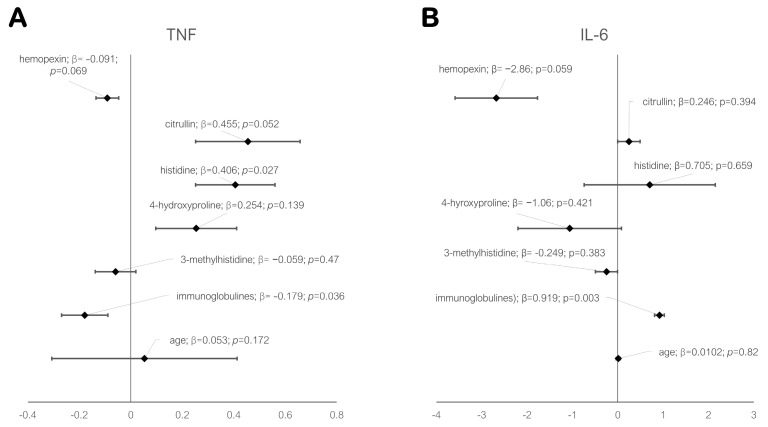
Forest Plot showing the regression coefficients of the confidence intervals of variables in a linear model testing the association of scores with response to anti-TNF (**A**) and anti-IL6 (**B**) therapy at week 24.

In order to determine significant indicators reflecting a positive response to therapy among patients, we tested by creating a multivariate regression model in which indicators such as 4-HP, 3-MH, hemopexin, total immunoglobulin fraction, citrulline, and arginine were tested in pairs, as well as the age factor (Figure 7). Adjustment for the significance of multiple testing was performed using the Bonferroni adjustment. Immunoglobulins (statistically significant, *p* = 0.036 for the anti-αTNF group and *p* = 0.003 for the anti-IL6 group) were positively associated with the effective treatment outcome in both study groups (Figure 7), indicating the reactivity of the immune system regardless of the chosen treatment scheme, as well as the generalized positive dynamics of the patients’ condition by the 24th week of treatment.

## 4. Discussion

### 4.1. Relation of anti-TNF and anti-IL6 Therapy to the Multiple Autoantibodies Inreconnection

The most distinctive amelioration in terms of objective and basic clinical indicators is observed among patients with anti-αTNF therapy, of which we previously noted a supreme level of immunoglobulins A and G compared to patients with anti-IL6 therapy (Table 2). We suggest that the presence of antibodies is a prerequisite for successful and effective therapy. Probably, the inspected rise of immunoglobulins level is due to the appearance of anti-citrullinated protein antibody (anti-CCP), and anti-CarP and anti-PAD4 antibodies, as an adaptive immune response. Antibodies against PAD4, citrullinated vimentin, and anti-CCP antibodies are found in 24%, 61%, and 74% of the RA patient population, respectively [14,15]. A positive correlation is found between disease duration and anti-PAD4 antibody titer. It should be noted that anti-PAD4 antibodies include A, G, and M isotypes, while anti-CCP antibodies include G isotypes.

Thus, the observed changes are probably based on the formation of immune complexes of antibodies with the corresponding antigens. This assumption is partly confirmed by previously obtained data on the level of TNF expression in patients with RA. Macrophages, as the main source of TNF producers, showed a significantly higher level of TNF secretion in response to the combined effect of anti-CCP antibodies and RF in comparison with the response to any one of these factors separately [16,17]. The reason for the rapid course of the disease and the deterioration of patients is a consequence of excessive production of TNF by macrophages under the condition of complex interaction with antibodies. A similar situation is observed in the case of IL-6, the production of which is noticeably higher under the complex action of anti-CCP antibodies or anti-PAD4 with RF. In contrast, patients who have either one of these types of antibodies as prevalent or seronegative patients generally have normal levels of TNF and IL6 secreted by synovial macrophages [18,19]. In the latter case, the activity of the disease is due to switching to other pro-inflammatory signaling pathways.

### 4.2. Does anti-TNF and anti-IL6 Therapy Have Even Chances to Cope with the Disease?

The scenario of the disease activity looks somewhat different between anti-IL6 and anti-TNF therapy. IL6 is a pleiotropic cytokine produced by numerous types of cells, including macrophages. In this regard, anti-IL6 therapy reduces inflammation and also has an anti-angiogenic effect. Blocking IL6 receptors usually demonstrates satisfactory therapeutic efficacy without significant toxicity in the treatment of RA [19,20]. Reduced secretion of C-reactive protein (CRP) is a reliable marker of the effectiveness of not so much anti-αTNF as anti-IL6 therapy (Figure 4). There is a positive correlation between CRP, anti-CCP antibodies, and IL6, as well as between these antibodies, CRP, and ESR. The suppression of CRP level is recognized after 2–4 weeks of treatment with anti-IL6 drugs in 90% of the treated population, and thereafter, an increase in hemoglobin levels in patients is observed after 3–8 weeks of treatment (Figure 4).

An improvement of patients can also be tracked through the increasing level of hemopexin, which is responsible for the transport of heme in the liver to its disposal site. In our study, hemopexin is an indirect indicator reflecting the change in the hemodynamic ESR showing a positive correlation (Figure 3). Proteomic data also indicate a recovery of hemopexin throughout the treatment period (24 weeks), which signifies a decrease in ESR as a result of inhibition of IL6-mediated inflammatory responses in patients.

However, an increase in hemopexin levels, in sense, may signify some types of anemia (in particular, hemolytic anemia), as well as chronic inflammatory processes caused by the high activity of cytokines and anaphylatoxins (complement factors), and suppression of immune reactivity [21].

Excessive synthesis of IL6 is also due to a complex of the above-mentioned circulating antibodies; however, treatment in most cases aims to block IL6 receptors but not to reduce the level of its secretion. There is evidence of a key contribution of IL6 to the development of the inflammatory response and a systemic effect on the activity of RA in comparison with TNF. Thus, IL6 is considered a cytokine that stimulates B-cells differentiation, and recent results indicate its role in the development of T-follicular helper cells, which is fairly strong evidence of the contribution of IL6 to the induction of plasma cells and the production of autoantibodies in patients with RA [22,23,24,25].

That is the reason why anti-IL6 therapy is used in some cases among patients who do not respond to anti-αTNF therapy, as TNF is stimulated by complement factors in response to anti-CCP antibodies [24,26,27]. Thus, both cytokines (IL6 and TNF) are not only a consequence of the developing inflammatory response, but also a trigger for the formation of citrullinated proteins and, thus, contribute to the formation of more citrullinated autoantigens through a positive loop.

One of the reliable markers of the effectiveness of anti-IL6 therapy is monitoring of the degradation of type III collagen, which is the main component of extracellular matrix that is destroyed by inflammatory cytokines, including IL6 [19,27]. The concept of multiple antibody types (multiple idiotypes) may explain why some of the patients may respond effectively to the certainly proposed strategy of therapy. Obviously, resistant patients are likely to have only one type of antibody. In these patients, disease activity is mediated by inflammatory signaling pathways unrelated to TNF production.

### 4.3. Essential Condition for the Successful Therapy

Data of immunochemical analysis indicate a correlation between the baseline level of antibodies in patients with RA and the baseline level of TNF and IL6 in the respective therapy groups. Changes in the level of antibodies, the level of TNF, and IL6 according to ELISA over 24 weeks of treatment show a correlation not only with objective indicators DAS-28 and CDAI but also with CRP and SRE. This correlation well reflects the possibility to predict the level of TNF and IL6 over time, and can be considered a promising prognostic indicator of the effectiveness of the response to anti-αTNF and anti-IL therapy to accomplish essential correction actions.

Currently, it seems that a high baseline titer of antibodies to RF, anti-CPP, and anti-PAD4 is most likely a condition for the successful implementation of anti-αTNF or anti-IL6 therapy. It seems on the surface that the complex of these antibodies is a prerequisite for the excessive production of cytokines by synovial macrophages. The response to treatment is manifested as an increased level of immunoglobulins due to the deployment of the adaptive response to switching from TNF signaling pathways and also the gradual secretion of anti-idiotypic antibodies. However, further observation of clinical parameters and changes in the proteome of patients for the following 24 weeks and post hoc analysis are required to frame the successful predictive model of the effectiveness of studied therapy schemes.

Clinical observations and biochemical studies show that among patients responding to anti-αTNF therapy, the effects of collagen resorption and bone resorption during treatment are quite often observed [28]. This is primarily due to compensatory excess synthesis of IL6 by fibroblast-like cells of synovial cells after treatment with anti-αTNF therapy, which, in turn, inhibits collagen synthesis by suppressing the α1-(I)-collagen gene promoter. Both cytokines have a suppressor effect on the promoter, but the activity of IL6 in relation to collagen is much more pronounced and specific than that of TNF [28]. In turn, TNF itself has an effect that stimulates the secretion of IL6.

### 4.4. Rebellion of Arginine

Arginine plays a key role in several metabolic pathways, but in the context of RA, we are interested in anabolic pathways regulated by the de novo arginine synthesis and catabolic pathways of arginine regulated by arginase and NO synthetase (NOS), as these pathways play an important role in inflammation. Arginine is synthesized by argininosuccinate synthetase (ASS) and argininosuccinate lyase (ASL) through the conversion of citrulline. Arginine catabolism is catalyzed by five different groups of enzymes: arginases (arginase-I and arginase-II) as part of the urea cycle, nitric oxide synthase (NOS for NO production), arginine decarboxylase, and arginine glycine amidinotransferase [29]. Through these pathways, arginine gives rise to ornithine, urea, polyamines, proline, NO, and citrulline [30]. Citrulline is the limiting factor in de novo arginine synthesis. The main precursor of citrulline is glutamine whereby this source makes up to 60–80% of all citrulline that enters the body [31]. The small intestine releases produced citrulline into the bloodstream, of which approximately 80% is taken up by the proximal tubular cells of the kidneys for de novo arginine synthesis. This pathway is known as the entero-renal axis of arginine metabolism. The balance between synthesis in the intestine and degradation in the kidneys determines the plasma concentration of citrulline. In addition, citrulline can also be obtained by converting ornithine by ornithine transcarbamoylase present in enterocytes and hepatocytes.

Arginine utilization is enhanced by the catabolic activity of arginase and NOS2 or increased protein synthesis in inflammatory conditions. Previously, an increase in NO synthesis was assumed in the initial phase of inflammation [29]. Based on elevated plasma nitrite and nitrate levels in patients with RA, and induced IL6 and TNF expression, the NOS2 releases more NO compared to other NOS enzymes. Thus, expectedly, NOS2 is crucial in hemodynamic changes during inflammation. Evaluation of increased NO synthesis as an inflammatory mediator leads to the idea of a violation of the catabolism of arginine and citrulline in patients with RA, as well as a deficiency or limited availability of arginine in such patients.

The immune response contributes to the depletion of arginine in the body. During inflammation, macrophages become active in response to a range of stimuli, including damaged cells and cytokines [32]. Non-activated macrophages show minimal arginine utilization and show no NOS2 or arginase activity. In the immune response, macrophages stimulate T-lymphocytes to produce Th1 or Th2 cytokines to maintain the predominant type of immune response [33,34]. It can be hypothesized that a decrease in arginine availability during inflammation is a protective mechanism to reduce excessive NO production by NOS2 synthase, as well as to regulate adaptive (T-lymphocyte) immune responses, preventing possible excessive inflammation and cytokine production. Arginase expressed by M2 macrophages is part of the anti-inflammatory response and competes with NOS2 synthase for arginine and, hence, contributes to arginine deficiency in M1/Th1 macrophages [33]. This leads not only to a decrease in macrophage NO production, but also to T cell dysfunction; however, it does not impair important aspects of T-cell function such as chemotaxis and cytotoxicity.

The most notable changes related to arginine metabolism were observed for 4-hydroxyproline (4-HP) and 3-methylhistidine (3-MH) throughout the observation of patients (Figure 5). Both metabolites, especially 4-HP, are important indicators of collagen regularization and maintenance of extracellular matrix and bone tissue. For example, the urinary level of 4-HP is generally within the normal range in patients with RA, but can be used as an indicator of joint tissue destruction and as a general sign of disease activity over a longitudinal study. However, patients of both anti-TNF and anto-IL6 groups displayed an increase in 4-HP at 12 weeks following stabilization of the level up to the 24th week. By contrast, the level of 3-MH altered unequally depending on the scheme of therapy. If patients with anti-IL6 therapy demonstrated a slow growth of 3-MH level during the entire treatment history, patients of the anti-TNF group were characterized by the increased level at the 12th week following the reduction at the end of monitoring. However, before the start of therapy, patients of the anti-TNF group had a more profound deficiency of arginine, which can explain the difference in alterations of 3MG between the groups of study.

The level of arginine is elevated in the synovial fluid of patients with RA and correlates with the level of the cytokine IL6 (Figure 7). Studies show that the inhibition of arginine uptake, such as with D-arginine, significantly inhibited its metabolism, cell proliferation, migration, and secretion of cytokines. Meanwhile, an arginine-deficient diet suppresses the progression of rheumatoid arthritis. Some studies show that replenishment of arginine deficiency by inhibiting arginine deaminase significantly reduces RA activity, while there is a significant decrease in serum IL6 concentration and a decrease in TNF gene expression, and the titer of anti-CPP.

Previously, we noted a decrease in disease activity in correlation with DAS28, CDAI, ESR, and CRP (Figure 2, Figure 3 and Figure 4). In the anti-TNF group of patients, a statistically significant correlation with a positive response was also found for histidine and citrulline (Figure 7), which is explained by the suppression of Th1 lymphocyte activity in these patients and, as a result, a decrease in NOS synthase activity. 

## 5. Conclusions

The study demonstrates that the level of immunoglobulins A and G increased sharply over 24 weeks of therapy in both anti-TNF and anti-IL-6 groups of patients but the anti-TNF strategy induced more severe alterations. In addition, µ-chains of immunoglobulins increased profoundly. Arginine metabolism was unequally affected between the groups of study. Initially, a severe deficiency of arginine was observed in groups of anti-TNF therapy compared to the anti-IL-6 patients before the start of therapy. Following 24 weeks, a significantly higher increase in arginine was monitored in anti-TNF groups. We found a positive effect on the level of restoration of the initial depleted arginine and the restoration of its catabolism on the level of citrulline, but the effect was much more explicit in the anti-TNF group compared to the anti-IL-6 subjects. In contrast, in the group of patients with anti-IL6 therapy, this indicator was characterized by a smoother gradual increase. Other indicators of bone tissue and collagen maintenance such as 4-HP and 3-MH behaved oddly in anti-TNF groups: following 12 weeks of gradual increase, the level of 4-HP decreased at the 24th week of monitoring, while in the anti-IL-6 group, the level of 4-HP smoothly increased over the complete monitoring time and increased significantly less compared to the anti-TNF group of patients. This indicates a different mechanism of matrix and bone tissue regularization between two groups of study, which is probably caused by an initial response of Th1 and Th2 macrophages, secreting depending on the arginine metabolism, its availability for interconversion, suppression of NOS2 activity, and further de novo synthesis of citrulline. The ELISA data also confirmed positive outcomes on both groups of study; however, patients who suffered anti-IL-6 demonstrated a more expeditive dynamic of IL-6 decrease and amelioration if considering indicators of disease severity and activity.

Ultimately, there was a positive correlation between indicators of severity of the disease (ΔCDAI, ΔDAS28 indicators) and ESR and CRP. The severity of the disease reduced as ESR and CRP decreased 20% and 40% compared to the baseline before treatment, respectively. However, hemopexin was closest to a positive correlation with the response and ESR; thus, a combination of these dynamic parameters played a more important role in the monitoring of treatment efficacy among patients of anti-IL6 therapy than among patients of anti-TNF therapy.

We assume that treatment with anti-IL6 therapy will be more effective in patients of whom RA is mediated by protein citrullination due to excessive activity of arginine deaminase. At the same time, anti-TNF therapy improves the ratio of arginine to ADMA (asymmetric dimethylarginine) in patients with inflammatory arthropathies by normalizing arginine catabolism and citrulline conversion in such patients.

## Figures and Tables

**Table 1:** Anthropometric, clinical, and psychometric characteristics of patients with schizophrenia and group of healthy volunteers participating in the study.

Parameter		Treatment Strategy	
		TNFi	IL6i
Sample size (n)		24	16
Disease duration, months		130.5 ± 129.4	157 ± 150
Anthropometric information	Male, %	21	27
	Age, means, years	54.0 ± 11.1	55.5 ± 11.8
	Height ± SD, cm	167.5 ± 7.4	167 ± 7.2
	Weight ± SD, kg	70.86 ± 13.87	70.3 ± 14.3
	Smokers, %	-	37
Clinical Information	Diagnosis: M05.8, %	86	64
	Diagnosis: M05.3, %	14	36
	CCP antibodies, positive, %	50	100
	Adalimumab, %	42	-
	Etanercept, %	29	-
	Dalibra, %	29	-
	Upadacitinib, %	-	-
	Sarilumab, %	-	100
Assessment of disease activity	Steinbroker	2.5 ± 0.8	2.6 ± 0.7
	CDAI ± SD	27.8 ± 9.3	29.4 ± 10.1
	DAS-28 ± SD	5.35 ± 0.95	5.4 ± 1.1
	HAQ ± SD	0.35 ± 0.44	0.06 ± 0.19
Biochemistry of blood	CRP, mg/mL	21.4 ± 26	22.4 ± 28.2
	ESR, mm/h	26.6 ± 15	23.6 ± 14.3

CCP—cyclic citrullinated peptide; CDAI score—clinical Disease Activity Index; DAS—disease activity score; HAQ score—health assessment questionnaire; ESR—erythrocyte sedimentation rate.

**Table 2:** Proteins most significantly influencing quantitative load over the course of therapy in anti-TNF and anti-IL6 therapy groups during 24 weeks of treatment (|FC| > 2, *p* < 0.05, adjusted by Bonferroni).

UniProt ID	Protein Name	Freq	FC	LOG FC	Biological Process
**anti-αTNF**					
P01871	Ig heavy constant mu	1	19.11	4.26	Adaptive immunity
A0M8Q6	Ig lambda constant 7	1	11.03	3.46	
P01877	Ig heavy constant alpha 2	1	7.84	2.97	
P0CG04	Ig lambda constant 1	1	7.82	2.97	
P01876	Ig heavy constant alpha 1	1	7.78	2.96	
B9A064	Ig lambda-like polypeptide 5	1	7.35	2.88	
P0DOY2	Ig lambda constant 2	1	6.81	2.77	
P0DOY3	Ig lambda constant 3	1	6.81	2.77	
P01860	Ig heavy constant gamma 3	1	5.97	2.58	
P0CF74	Ig lambda constant 6	1	5.73	2.52	
P01861	Ig heavy constant gamma 4	1	5.54	2.47	
P19652	Alpha-1-acid glycoprotein 2	1	5.38	2.43	Acute phase
P01023	Alpha-2-macroglobulin	1	5.13	2.36	
P01024	Complement C3	1	4.94	2.31	Complement alternate pathway
P01009	Alpha-1-antitrypsin	1	4.92	2.30	Acute phase
P01857	Ig heavy constant gamma 1	1	4.43	2.15	Adaptive immunity
P02790	Hemopexin	1	4.20	2.07	Transport
P02765	Alpha-2-HS-glycoprotein	1	4.17	2.06	Mineral balance
P00739	Haptoglobin-related protein	0.8	0.25	−2.01	Acute inflammatory response
**anti-IL6**					
P01009	Alpha-1-antitrypsin	1	0.34	−1.37	Acute phase
P01023	Alpha-2-macroglobulin	0.87	2.2	1.1	
P01024	Complement C3	0.57	2.7	1.4	Complement alternate pathway
P02765	Alpha-2-HS-glycoprotein	0.57	3.4	1.8	Mineral balance

## Data Availability

The data obtained throughout the experiments can be provided by A.L.K. upon reasonable request.
